# Responsible Use of Artificial Intelligence Tools in Imaging Reporting: Practical Guidelines for Sonographers—A Narrative Review

**DOI:** 10.3390/healthcare14203548

**Published:** 2026-10-21

**Authors:** Mozdalifah Elnaeem

**Affiliations:** Diagnostic Radiography Technology Department, College of Nursing and Health Sciences, Jazan University, Jazan 45142, Saudi Arabia; mmelnaem@jazanu.edu.sa

**Keywords:** artificial intelligence, sonographer, ultrasound, imaging reporting, clinical guidelines, automation bias, human oversight, large language models, reporting standards, patient safety

## Abstract

**Highlights:**

**What are the main findings?**

- AI now supports the entire ultrasound reporting chain, from real-time acquisition guidance (diagnostic-quality images in 98.8% of novice-obtained echocardiographic scans) to computer-aided diagnosis (AUC improving from 0.83 to 0.87 in breast lesion assessment) and large-language-model report drafting.
- Automation bias, deskilling, hallucinated content, algorithmic bias, and unresolved medico-legal accountability are the principal risks of uncritical reliance on AI outputs.

**What are the implications of the main findings?**

- A phase-based good-practice framework—appraisal and training before use, verification during use, and documentation and audit after use—offers sonographers a practical route to safe AI adoption.
- AI should augment, not replace, sonographer judgment; human oversight, verification of every AI output, and continuing AI literacy are prerequisites for its use in imaging reporting.

**Abstract:**

Artificial intelligence (AI) is rapidly entering diagnostic ultrasound, supporting image acquisition, automated measurement, computer-aided diagnosis, and report drafting. Sonographers are primary users of these tools, yet sonographer-oriented guidance on their responsible use in imaging reporting remains fragmented. This narrative review synthesizes current evidence, reporting guidelines, trustworthiness frameworks, regulations, and professional standards relevant to AI-assisted imaging reporting to distill practical guidance for sonographers. PubMed/MEDLINE, Scopus, and Google Scholar were searched, together with professional-body and regulatory documents published up to July 2026. The review maps AI applications across the reporting chain, from real-time acquisition guidance (AI-guided echocardiography achieving diagnostic-quality images in 98.8% of novice-obtained scans) to computer-aided diagnosis improving breast lesion assessment (area under the curve increasing from 0.83 to 0.87) and large language models drafting structured reports. Evidence on automation bias, deskilling, hallucinated content, algorithmic bias, and medico-legal accountability is critically appraised. Building on FUTURE-AI, CONSORT-AI, DECIDE-AI, and World Health Organization guidance, a phase-based good-practice framework is proposed, covering pre-use appraisal and training, verification during use, and documentation and audit after use. AI should augment, not replace, sonographer judgment; human oversight, verification of every AI output, transparent documentation, and continuing AI literacy are prerequisites for safe adoption in imaging reporting.

## 1. Introduction

Diagnostic medical ultrasound is one of the most widely used imaging modalities worldwide: it is inexpensive, portable, free of ionizing radiation, and uniquely real-time. It is also the most operator-dependent cross-sectional modality, because image quality and diagnostic content depend directly on the skill of the person holding the transducer—most often a sonographer. Over the past decade, artificial intelligence (AI), and particularly deep learning, has moved from research prototypes to everyday clinical tools embedded in ultrasound systems, promising to standardize acquisition, automate measurements, support interpretation, and accelerate reporting [1,2,3]. Surveys of deep-learning applications in ultrasound now span obstetrics, cardiology, breast, thyroid, abdominal, and musculoskeletal imaging, and dedicated reviews document an exponential growth of AI research in fetal ultrasound and image analysis more broadly [3,4]. Regulatory activity mirrors this expansion: the United States Food and Drug Administration (FDA) maintains a public list of authorized AI/machine learning (ML)-enabled medical devices that surpassed 1400 entries by the end of 2025 (list accessed 17 July 2026; the count continues to rise), with radiology consistently representing the largest specialty, and the European Union’s Artificial Intelligence Act—Regulation (EU) 2024/1689—classifies most AI used in medical devices as high-risk, imposing explicit requirements for human oversight [5,6].

Within this changing ecosystem, the imaging report remains the definitive, legally relevant product of an ultrasound examination. AI now touches every step that leads to that report: guidance software that coaches the operator toward diagnostic-quality views; algorithms that auto-measure fetal biometry or ejection fraction; computer-aided detection and diagnosis (CAD) systems that stratify lesions into BI-RADS or TI-RADS categories; and, most recently, large language models (LLMs) that transform findings into structured draft reports. International organizations have responded with ethical and governance frameworks—the World Health Organization (WHO) issued guidance on the ethics and governance of AI for health in 2021 and dedicated guidance on large multi-modal models in 2024, explicitly warning against automation bias and unsupervised delegation of clinical judgment [7,8]. Editorials in the ultrasound literature similarly call for “responsible stewardship,” urging professional societies to define implementation, validation, and scope-of-practice boundaries for AI in sonography [9].

A striking gap persists, however. Most AI guidance documents are written for developers, researchers, or interpreting physicians. Sonographers—who acquire the images, interact with AI outputs in real time, verify measurements, and in many jurisdictions author or co-author the technical content of reports—rarely appear as the primary audience. Because the quality and integrity of every downstream AI output depends on the human at the scanner, the sonographer is, in effect, the first and most consequential checkpoint in the AI-assisted reporting chain. Misuse at this checkpoint—accepting an incorrect measurement, following a wrong CAD suggestion, or signing off unverified AI-drafted text—propagates directly into the clinical record and can harm patients.

The aim of this narrative review is therefore threefold: (1) to map how AI tools are currently used across the ultrasound imaging reporting pathway, with emphasis on evidence relevant to sonographer practice; (2) to synthesize the current landscape of guidelines, frameworks, regulations, and professional standards that govern—or should govern—AI use in imaging reporting; and (3) to translate this evidence into practical, phase-based good-practice guidelines for sonographers, supported by recommendations for education, institutional governance, and future professional-society action. The review is intended for practicing sonographers, sonography educators, ultrasound department managers, and policy makers concerned with the safe integration of AI into diagnostic imaging services.

## 2. Literature Search and Scope of the Review

This article is a narrative review, chosen because the research questions span heterogeneous sources—original research, consensus guidelines, regulatory documents, and professional standards—that cannot be meaningfully pooled with systematic methodology. A structured search of PubMed/MEDLINE, Scopus, and Google Scholar was performed on 18 July 2026 for literature published from January 2015 to July 2026. Database-specific strings combined three concept blocks with Boolean operators: the technology (“artificial intelligence,” “deep learning,” “machine learning,” “large language model”), the modality and profession (“ultrasound,” “sonography,” “sonographer”), and the reporting context (“imaging reporting,” “structured reporting,” “automation bias,” “deskilling,” “guideline”). The complete database-specific search strings, filters, search dates, and numbers of records retrieved per database are provided in Supplementary Table S1. Google Scholar results were screened to the first 200 records per query, sorted by relevance. Searches were supplemented by targeted review of documents issued by professional bodies (American Institute of Ultrasound in Medicine (AIUM), World Federation for Ultrasound in Medicine and Biology (WFUMB), European Federation of Societies for Ultrasound in Medicine and Biology (EFSUMB), International Society of Ultrasound in Obstetrics and Gynecology (ISUOG), Society and College of Radiographers/British Medical Ultrasound Society), regulatory agencies (United States FDA, European Union), and the WHO, identified through their official websites. Reference lists of key papers were hand-searched for additional sources.

Eligibility was pragmatic. Included were English-language publications reporting (a) original evaluations of AI tools used in ultrasound acquisition, measurement, interpretation, or reporting; (b) reporting guidelines and quality-assessment tools for AI research in medical imaging; (c) ethics, governance, or trustworthiness frameworks for health AI; (d) regulations and regulatory decisions for AI-enabled imaging devices; and (e) professional standards describing the sonographer’s role in examination documentation and reporting. Editorials and expert commentaries from high-impact journals were included where they articulated professional positions not yet captured in formal guidelines. Sources were excluded if they concerned AI applications outside medical imaging, were not available in English, or were superseded by a more recent version of the same guideline. Regulatory and professional-organization documents are cited with their access dates because their content evolves over time.

Records were exported to a reference manager, and duplicates were removed automatically and then confirmed manually. Titles and abstracts, and subsequently full texts, were screened against the eligibility criteria by the sole author; no second reviewer was involved, and no formal inter-rater agreement could therefore be assessed. In total, 2506 records were retrieved across the three databases (PubMed/MEDLINE 612; Scopus 894; Google Scholar 1000 screened), 1714 remained after duplicate removal, 126 full texts were assessed, and 43 sources (including 7 identified through professional-body and regulatory websites and 2 through hand-searching) were retained for synthesis. Selection was purposive rather than exhaustive: for each theme, primary validation studies were preferred over secondary reviews or regulatory summaries for quantitative performance claims, the most recent version of each guideline or regulation was retained, and ultrasound-specific evidence was preferred over evidence from other imaging modalities, which was used only where ultrasound-specific evidence was lacking and is explicitly labeled as extrapolated wherever it is cited.

The synthesis is organized thematically. Section 3 situates the sonographer’s role in imaging reporting; Section 4 reviews AI applications along the reporting chain with their supporting evidence; Section 5 summarizes the guideline and regulatory landscape; Section 6 critically examines the principal risks; Section 7 distills the evidence into good-practice recommendations, including operational verification rules and a categorization of each recommendation by evidentiary basis and responsible level; Section 8 addresses education and implementation; Section 9 states the limitations of this review and future directions. Because this is a narrative rather than a systematic review, no formal risk-of-bias assessment was undertaken; instead, the strengths and limitations of the evidence are discussed qualitatively where relevant, quantitative results are reported with their original effect sizes, study design, population, and comparator to allow readers to judge the strength of each claim, and each recommendation in Section 7 is labeled according to whether it rests on a regulatory or professional requirement, a consensus framework, direct ultrasound evidence, extrapolated evidence, or author judgment. The consequences of this methodological choice for selection bias and evidentiary strength are discussed in Section 9.

## 3. The Sonographer’s Role in Imaging Reporting

The sonographer’s contribution to the imaging report begins long before any text is written. Professional standards consistently frame the sonographer as the guarantor of image adequacy and technical documentation. The AIUM Practice Parameter for Documentation of an Ultrasound Examination requires a permanent, retrievable record of every examination, with images labeled by patient identifiers, facility, date, anatomic location, and orientation, and it specifies the required elements of the final signed report—including the indication, a description of findings using standard terminology, an impression, comparison with prior studies, and documentation of any significant discrepancy between preliminary and final reports [10]. Accreditation standards of the same organization define personnel requirements, mandating recognized certification for sonographers (for example, through the American Registry for Diagnostic Medical Sonography or the American Registry of Radiologic Technologists), quality-assurance programs, and timely availability of the signed final report [11]. European bodies frame sonographer activity similarly: EFSUMB explicitly directs its clinical practice guidelines at “physicians and sonographers” performing or requesting ultrasound examinations [12], and in the United Kingdom, joint professional working standards of the Society and College of Radiographers and the British Medical Ultrasound Society recognize the reporting sonographer as an established advanced role in which appropriately trained sonographers issue autonomous reports within defined scopes of practice [13].

Two features of this role are directly relevant to AI governance. First, sonography is a craft of real-time judgment: the sonographer continuously decides which anatomy to display, how to optimize the image, which measurements to take, and what to record for the interpreting provider. Each of these decisions determines the input data on which any AI tool operates; an AI system fed a suboptimal or wrongly labeled view will produce a confident but wrong output, a failure mode often summarized as “garbage in, garbage out.” Second, accountability for the report is legally and professionally anchored in named humans. The allocation of that accountability, however, is jurisdiction-specific. In the United States and many other health systems, the interpreting physician signs the definitive report, while the sonographer is accountable for the technical adequacy and fidelity of the acquired record; in the United Kingdom and some other systems, appropriately credentialed reporting sonographers issue autonomous reports within a defined scope of practice and hold direct sign-off responsibility [10,13]; in other countries, sonographer reporting is not recognized at all. Throughout this review, statements about who signs, who is accountable, and what must be documented should therefore be read as illustrative of these models rather than as universally applicable, and readers should apply the professional and legal requirements of their own jurisdiction. No framework currently transfers any share of this accountability to software, and medico-legal analyses consistently locate responsibility with the human operator and supervising clinician [9]. Any guideline for AI use in reporting must therefore start from the premise that AI outputs are provisional inputs to human judgment, not autonomous contributions to the record.

It follows that the sonographer’s role in the AI era is not diminished but reframed. Beyond producing diagnostic-quality images, the sonographer becomes (a) a verifier of AI-generated measurements and labels against the live image; (b) a gatekeeper who ensures that AI tools are used only within their cleared intended use and by appropriately trained staff; (c) a documenter who records when and how AI contributed to the examination; and (d) a participant in local audit and quality improvement of AI performance. These expanded responsibilities echo the stewardship agenda articulated in the WFUMB literature, which calls for profession-led implementation guidance, scope-of-practice boundaries, and training that preserves unaided competence while adding AI literacy [9]. Section 4, Section 5 and Section 6 build the evidence base for this reframing before Section 7 converts it into operational recommendations.

## 4. AI Tools Across the Ultrasound Imaging–Reporting Chain

### 4.1. AI-Assisted Image Acquisition and Quality Control

AI enters the reporting chain at the earliest possible point: the acquisition of the images on which the report will be based. The landmark regulatory event in this domain was the 2020 De Novo authorization of Caption Guidance (Caption Health), the first FDA-authorized software providing real-time AI guidance for medical imaging acquisition [5,14]. The system emulates expert sonographer guidance, issuing prescriptive instructions on transducer manipulation, displaying a real-time image-quality meter, and auto-capturing clips judged to be of diagnostic quality. In the pivotal prospective study [15], conducted at two hospitals in the United States with a single ultrasound platform, eight registered nurses with no prior ultrasound experience, after a short training course, used the software to acquire limited ten-view transthoracic echocardiograms in 240 adult patients; a blinded panel of expert echocardiographers judged the scans adequate for qualitative assessment of left ventricular size, left ventricular function, and pericardial effusion in 98.8% of cases and of right ventricular size in 92.5% [15]. Several features of this study qualify the 98.8% figure. The endpoint was image adequacy as judged by experts for a limited set of qualitative questions, not diagnostic accuracy, time to diagnosis, or patient outcome; the acquisition protocol was a limited study rather than a comprehensive echocardiogram; the operators, setting, and equipment were homogeneous; and the study was designed to support regulatory authorization of the device [14]. The result therefore establishes technical feasibility of AI-guided acquisition by non-experts under study conditions and should not be read as evidence that guided novices can replace sonographers, or that clinical outcomes improve, in routine practice. Performance was weaker for some secondary targets—83.3% for tricuspid valve structure and only 57.5% for inferior vena cava size—illustrating that even well-validated acquisition AI has view-specific limitations that users must know [14,15]. Independent validation work continues in this area, including a deep-learning modular prototype that successfully guided inexperienced operators to acquire diagnostic-quality ultrasound images of the urinary system [16].

For sonographers, acquisition AI has two practical implications. On one hand, real-time quality feedback can function as a training accelerant and a consistency tool, narrowing the gap between operators and supporting task-shifting in settings with workforce shortages. On the other hand, the labeled intended use matters: these systems are cleared for specific views, patient populations, and user categories, and the presence of an automated quality meter does not certify that a view is clinically adequate for every diagnostic question. The sonographer remains responsible for confirming that the acquired views answer the clinical indication, for recognizing situations—obesity, unusual anatomy, pediatric patients—in which the algorithm may degrade, and for documenting any limitation of the examination, as required by documentation standards [10,15].

### 4.2. Automated Measurements and Quantification

Automated quantification is the most mature and most deeply embedded class of ultrasound AI. Commercial systems now offer one-click or continuous automated measurement of fetal biometry, cardiac ejection fraction and strain, nodule dimensions, and Doppler indices, and regulators have cleared obstetric measurement tools (for example, deep-learning software for fetal biometry on handheld devices) and fetal anomaly detection support within the FDA’s AI/ML device list [3,4,5]. Reviews of fetal ultrasound AI document steady progress in standard-plane detection, structure segmentation, and automated biometry, while cautioning that most models are validated retrospectively on curated datasets rather than prospectively in diverse clinical populations [4]. Automated ejection fraction computation, paired with guided acquisition, is marketed as a way to standardize a measurement notorious for inter-observer variability [14].

The reporting relevance is direct: measurements populate the quantitative core of the report, and errors here are numeric, plausible-looking, and therefore dangerous. Best practice—consistent with the traceability and quality-control recommendations of international AI frameworks—is to treat every automated measurement as a preliminary value: the sonographer should visually confirm that calipers are placed on the correct anatomic landmarks, that the correct cardiac phase or fetal plane was used, and that values are physiologically plausible before they are committed to the report, re-measuring manually whenever the automated placement is doubtful [10,17]. Where software versions change, departments should re-verify measurement behavior, because algorithms can be updated under predetermined change control plans that alter performance characteristics between software versions [5].

### 4.3. Computer-Aided Detection and Diagnosis

CAD systems represent the most clinically visible AI category in ultrasound reporting, with the strongest evidence base in breast and thyroid imaging. In breast ultrasound, the retrospective multireader, multicase study of Koios Decision Support—900 lesions with known outcomes read by 15 physicians with and without the AI output—found that mean reader AUC improved from 0.83 with ultrasound alone to 0.87 with AI decision support, while inter-observer agreement improved from 0.54 to 0.68 and intra-observer variability fell from 13.6% to 10.8% [18]. The absolute AUC gain of 0.04 is modest, was obtained under experimental reading conditions without the live examination or clinical context, and describes reader-level discrimination rather than patient outcomes; its principal clinical relevance is the reduction in variability between and within readers, which is itself a technical rather than an outcome measure. The S-Detect system showed, in a multicenter prospective study of 757 masses detected in an opportunistic-screening population, significantly higher AUC (0.83 versus 0.74) and specificity (74.35% versus 54.13%) than radiologists reading conventional ultrasound, without loss of sensitivity, with histopathology or follow-up as the reference standard [19]; a meta-analysis of ten studies reported pooled sensitivity of 0.82 and specificity of 0.86 for differentiating benign from malignant masses [20]. Potentially consequential effects on management have also been modeled: in a retrospective analysis of BI-RADS 4A lesions assessed as “possibly benign” on both orthogonal images, applying the AI output would have reduced the biopsy rate from 100% to 67.4% without missing cancers, whereas relying on a single benign image assessment would have reduced biopsies further but missed two cancers—an instructive demonstration that the value of CAD depends on how conservatively its output is used [21]. This was a simulation on archived cases; a prospective reduction in benign biopsies or missed cancers attributable to CAD in routine ultrasound practice has not yet been demonstrated. In thyroid imaging, data summarized in a review of FDA-cleared tools for an AI risk-stratification system reported an AUC of 0.80 and mean reader improvements in AUC, sensitivity, and specificity when the tool was used; these figures derive from regulatory submissions rather than independent peer-reviewed validation, and external validation remains limited [22]. A broader clinical review of breast ultrasound AI reaches a consistent conclusion: current tools function best as a second reader that reduces variability, with benefits that vary by reader experience [23].

Across these studies, it is important to distinguish technical performance and stand-alone or reader-level diagnostic accuracy—which is what the evidence base currently demonstrates—from improvements in clinical outcomes, workflow efficiency, or patient safety, for which prospective evidence in ultrasound is still lacking. Three reporting-relevant lessons emerge. First, CAD output is probabilistic decision support, not a diagnosis; it must be integrated with the live real-time examination, clinical context, and prior imaging rather than transcribed into the report. Second, the magnitude of benefit is experience-dependent—junior readers gain sensitivity, while experienced readers may gain little or even lose specificity—so the same tool can help one sonographer–radiologist team and hinder another [18,23,24]. Third, CAD performance is conditional on input quality and population match: systems trained and validated on selected images from particular vendors and institutions may degrade on different equipment or demographics, a generalizability constraint emphasized across reviews [4,23].

### 4.4. Automated and Generative Report Drafting

The newest link in the chain is the use of LLMs and related generative models to produce or reformat report text. The available evidence must be read according to what each study type can and cannot establish. Feasibility studies show that GPT-4-class models can transform free-text radiology findings into structured reports across languages with high fidelity to the source content [25]; this demonstrates technical feasibility on curated inputs, not clinical reporting safety. Retrospective evaluations show that such models can detect factual and interpretive errors deliberately inserted into radiology reports with useful accuracy [26]; this is a retrospective benchmark, not evidence of error reduction in live reporting. Benchmark performance approaching the passing threshold on radiology board-style examinations [27] indicates knowledge recall on multiple-choice questions and should not be interpreted as readiness for clinical report generation. None of these studies involved ultrasound reports, sonographers, or prospective clinical use, and demonstrated clinical utility—fewer reporting errors, faster turnaround, or better patient outcomes with LLM-assisted ultrasound reporting—has not been shown. Practical primers on chatbots and LLMs in radiology emphasize realistic near-term roles—drafting structured impressions, converting narrative text to templates, summarizing comparison studies, and improving report readability—while stressing that these systems generate fluent text without genuine understanding and can fabricate content, a failure mode known as hallucination [28]. WHO guidance on large multi-modal models likewise highlights the risk of false, inaccurate, biased, or incomplete statements and explicitly cautions against automation bias in professional use [8].

For sonographers and reporting teams, generative drafting is simultaneously the most convenient and the most hazardous AI application. The convenience is real: standardized phrasing, fewer typographical errors, and time savings in high-volume lists. The hazard is subtle wrongness—an invented measurement, a transposed laterality, a plausible-sounding but unsupported impression—that can survive superficial proofreading precisely because the text reads well. Regulatory recognition of generative reporting tools is only beginning, and professional documentation standards already require that the signed report accurately reflect the examination actually performed [5,10]. The operative rule, developed further in Section 7, is that AI-drafted text must be verified line by line against the images and measurements before sign-off, and that no finding, measurement, or recommendation should enter the report solely because a model generated it [8,25,28]. Table 1 summarizes representative AI applications across the chain together with their evidence and practical cautions.

## 5. The Current Guideline Landscape Relevant to Sonographers

### 5.1. Reporting and Evaluation Guidelines for AI Research

A family of consensus guidelines now standardizes how AI research should be reported and evaluated, and these documents—though aimed at researchers—equip sonographers to appraise vendor claims and study results critically. The Checklist for Artificial Intelligence in Medical Imaging (CLAIM) specifies the minimum information that studies of AI in medical imaging should report, including data provenance, ground-truth definition, and evaluation design [29]. TRIPOD + AI provides updated guidance for reporting clinical prediction models that use regression or machine learning, requiring transparent description of data sources, participants, predictors, and performance [30]. For diagnostic accuracy studies specifically, the STARD-AI initiative is developing an AI-centered extension of the Standards for Reporting of Diagnostic Accuracy Studies [31], complemented by QUADAS-AI, a quality-assessment tool for judging risk of bias in AI-centered diagnostic accuracy research [32]. For clinical trials of AI interventions, the SPIRIT-AI and CONSORT-AI extensions add fifteen and fourteen AI-specific items, respectively, to the standard protocol and trial reporting checklists—including algorithm version, input-data handling, human–AI interaction, and analysis of error cases [33,34]. DECIDE-AI addresses the crucial intermediate stage of early, small-scale deployment in live clinical settings, requiring reporting of human factors, user characteristics, workflow integration, and reasons for deviation from AI recommendations [35].

Why should a sonographer read research-reporting guidelines? Because the questions these checklists formalize are precisely the questions a user should ask of any AI tool placed on a scanner: On what data was it trained and validated? Was the validation external, multi-site, and prospective, or internal and retrospective? Which version is installed, and how are updates handled? What level of human interaction did the evidence assume, and with what training? Tools supported only by retrospective, single-center, internally validated studies carry a wider margin of uncertainty than their marketing suggests; guidelines such as CLAIM, TRIPOD + AI, and DECIDE-AI give department managers and sonographer leads a concrete appraisal vocabulary for procurement and local validation decisions [29,30,35].

### 5.2. Trustworthiness and Ethics Frameworks

Above study-level reporting sit overarching frameworks for trustworthy health AI. The FUTURE-AI guideline—developed by a consortium of 117 interdisciplinary experts from 50 countries—defines six guiding principles (fairness, universality, traceability, usability, robustness, explainability) and thirty best practices spanning design, development, validation, regulation, deployment, and monitoring [17]. Several FUTURE-AI practices translate almost verbatim into sonographer duties: continuous quality control of AI inputs and outputs, provision of calibrated uncertainty information, periodic auditing for performance drift, logging of AI predictions and clinical decisions, and user training matched to the diversity of end users [17]. The WHO 2021 guidance established six ethical principles for health AI, including protecting human autonomy, promoting safety, ensuring transparency and explainability, fostering accountability, and ensuring equity [7]; its 2024 companion guidance on large multi-modal models added more than forty recommendations and explicitly warned that such models can encourage automation bias among health professionals [8]. The European Society of Radiology’s white paper remains a useful imaging-specific orientation to data, validation, and workflow questions [36], and the widely cited “20 critical questions” framework offers a rapid critical-appraisal scaffold for transparency, replicability, ethics, and effectiveness [37].

These frameworks converge on one message for the reporting room: trust in AI must be earned continuously at the point of use, not assumed from regulatory clearance or vendor claims. Clearance establishes a floor of safety and effectiveness for a defined intended use; it does not guarantee performance in a different population, on different equipment, or after software updates. Sonographers contribute directly to the monitoring that frameworks such as FUTURE-AI require—by recognizing and reporting implausible outputs, participating in local audit, and feeding discrepancies back into departmental governance [17].

### 5.3. Regulatory and Professional Governance

Regulation defines the outer boundary of permissible use. In the United States, most imaging AI reaches the market through the FDA’s 510(k) pathway, with novel device types such as AI-guided acquisition authorized through the De Novo pathway with special controls; the agency’s public AI/ML device list documents each authorization and increasingly accommodates algorithm updates through predetermined change control plans [5,14]. Analyses of authorizations repeatedly note, however, that pivotal evidence is often retrospective and that demographic and training-data transparency in decision summaries is incomplete—reinforcing the need for local due diligence beyond the fact of clearance [5]. In the European Union, the Artificial Intelligence Act designates AI components of medical devices as high-risk systems, imposing obligations for risk management, data governance, technical documentation, transparency, human oversight, accuracy, and post-market monitoring; Article 14 specifically requires that high-risk AI be designed so that natural persons can effectively oversee it, including the ability to interpret outputs, recognize automation bias, and override or interrupt the system [6]. WHO guidance assigns governments primary responsibility for regulating health AI and calls for mandatory post-release auditing of widely deployed models [8].

Professional governance is catching up more slowly. Ultrasound-specific standards—AIUM practice parameters and accreditation requirements, EFSUMB policy documents, and joint working standards for sonographers—define documentation, quality-assurance, personnel, and scope-of-practice expectations but have only begun to address AI explicitly [10,11,12,13]. A 2026 WFUMB editorial is the most direct call to action, arguing that professional societies should issue guidance on AI implementation, validation, and scope-of-practice boundaries; that publication standards should demand prospective multi-site validation and subgroup performance reporting; that training must preserve unaided competence while adding AI literacy; and that manufacturers and regulators must ensure transparent monitoring and disclosure of limitations [9]. Until such ultrasound-specific guidance matures, sonographers and their departments must operationalize the general frameworks reviewed here. Table 2 summarizes the principal guidelines and frameworks and their relevance to sonographer practice.

## 6. Key Risks of AI Use in Imaging Reporting

### 6.1. Automation Bias and Over-Reliance

Automation bias—the tendency of humans to favor machine-generated suggestions and to omit contradictory checks—is the most thoroughly documented risk at the human–AI interface in imaging. It should be stated clearly that the controlled experimental evidence summarized in this section comes from mammography, chest radiography, and pathology, not from ultrasound; no comparable experiment has yet exposed sonographers to deliberately incorrect AI suggestions during real-time scanning, and the findings below are therefore extrapolated to sonographer workflows. In a randomized experiment on mammography, readers of varying experience were exposed to a CAD system that, for a subset of cases, deliberately gave an incorrect BI-RADS suggestion; when the suggestion was wrong, diagnostic accuracy collapsed from roughly 80% under correct advice to 19.8% among inexperienced readers, 24.8% among moderately experienced readers, and 45.5% even among very experienced readers, despite the underlying images being identical [38]. Comparable susceptibility has been demonstrated for chest radiography, where clinicians followed incorrect AI advice even when told that the system was unreliable, with effects extending to experts [39], and in pathology, where a deep-learning assistant improved overall performance but induced readers to follow incorrect suggestions on hard cases [40]. Crucially, the mammography experiment also documented discriminative automation bias—readers were more likely to accept an incorrect AI suggestion that downgraded a lesion to benign than one that upgraded it—showing that the direction of error can align with what is most convenient or most reassuring [38].

The reporting implications are sobering, with the caveat that their direct generalizability to ultrasound is uncertain: real-time scanning differs from static image review because the operator can re-acquire views and re-measure, which may reduce susceptibility, but it also adds time pressure and cognitive load, which may increase it. The only ultrasound-specific signal is indirect—the experience-dependent benefit and susceptibility observed in breast-ultrasound CAD reader studies [18,24]. An incorrect automated measurement, an implausible CAD category, or a wrong LLM-drafted impression is most dangerous when it is plausible and when the reader is time-pressured—the default conditions of many ultrasound lists. Defenses are behavioral and organizational rather than technical: deliberate verification routines (checking every AI-derived value against the image), report templates that force review of AI-flagged items, audit of cases where users overrode or accepted AI advice, and a departmental culture in which questioning the algorithm is expected rather than exceptional [17,38]. Regulation reinforces these defenses: EU AI Act Article 14 makes effective human oversight—including the ability to recognize and counteract automation bias—a legal design requirement for high-risk systems [6], and WHO guidance lists automation bias among the primary professional risks of large multi-modal models [8].

### 6.2. Deskilling and Erosion of Core Competencies

A subtler, slower risk is the erosion of the manual skills that make verification possible. If trainees learn anatomy and measurement only through AI-assisted workflows, they may never acquire the unaided competence needed to recognize when the AI is wrong—the very skill on which safe oversight depends. Ultrasound-specific evidence from breast CAD reader studies shows that the presence and value of AI support interact with reader experience, with junior readers deriving the largest sensitivity gains [18,24]; the greatest susceptibility of inexperienced readers to incorrect suggestions has been demonstrated in mammography [38] and is extrapolated here. Direct evidence of deskilling in sonographers is absent; the concern that prolonged reliance on decision support reduces vigilance and independent diagnostic performance over time derives from reader studies in other modalities and from human-factors theory [24,38], and quantifying deskilling trajectories in sonography is identified as a research priority in Section 9. The WFUMB stewardship editorial makes the training requirement explicit: curricula should preserve unaided scanning and interpretive competence while deliberately adding AI literacy, so that every operator retains the ability to function safely when the tool is unavailable, out of scope, or simply wrong [9].

For sonography departments, the practical countermeasures include staged exposure—manual measurement and unaided scanning remain mandatory in basic training, with AI tools introduced after core competencies are demonstrated—periodic unaided practice and competency assessment, and inclusion of “AI-off” scenarios in continuing professional development. These measures align with accreditation expectations that personnel competency be maintained and documented, and they protect patients against the failure modes that only a skilled human can catch [9,11].

### 6.3. Hallucinated or Erroneous Content in Generative Reporting

Generative models introduce a failure mode distinct from classical CAD: fluent fabrication. LLMs can produce measurements, comparisons, or impressions that were never observed, because their objective is plausible text rather than verified fact; WHO guidance warns that outputs can be false, inaccurate, biased, or incomplete and stresses the need for human verification [8]. Empirically, the same technology that drafts reports can also police them—a study of GPT-4 found useful performance in detecting errors in radiology reports—suggesting a future pattern of machine drafting plus machine checking under human sign-off [26]. Drafting performance in structured-report conversion has been shown to preserve source content well, but these studies are feasibility demonstrations with curated radiology inputs, not safety evidence for unsupervised use, and none has been conducted on ultrasound reports or with sonographers as users [25,26].

The operational rules follow directly. AI-generated text must be treated as an unverified draft: every numerical value must match the measured record, every finding must correspond to the images, laterality and units must be checked, and any comparison with prior studies must be confirmed against the actual prior report. Departments should prohibit direct insertion of model output into signed reports without human review, should prefer retrieval-grounded, template-constrained implementations over free generation, and should record in local policy which drafting tools are approved, for which examination types, and under what review conditions [8,10,28].

### 6.4. Algorithmic Bias, Generalizability, and Equity

AI performance is not uniform across patients. Models inherit the biases of their training data, and clinical-safety analyses have long warned that algorithmic bias can systematically disadvantage under-represented groups [41]. Imaging-specific concerns include models trained predominantly on particular vendors’ equipment, on single-institution populations, or on demographic mixes unlike the deployment site; validation studies frequently under-report demographic composition and subgroup performance, and external, multi-site validation remains the exception rather than the rule [5,17,22]. FUTURE-AI elevates fairness and universality to first-class principles, requiring bias-aware data practices, subgroup performance reporting, and monitoring across diverse populations [17], and reporting guidelines now explicitly demand the demographic and site transparency that makes generalizability assessable [29,30].

Sonographers encounter this risk at the bedside: a CAD tool validated on adult outpatients may misclassify pediatric lesions; an automated biometry tool trained on one population may bias growth assessments in another; an LLM drafting reports may handle some languages or dialects better than others. The appropriate professional response is heightened, not reduced, vigilance for patients who differ from the typical validation population, plus systematic reporting of suspect outputs so that local experience accumulates into governance. Encouragingly, well-designed studies also show equity-promoting potential: an AI-supported triage study in a low-resource setting demonstrated that appropriately validated tools can extend expert-level breast assessment where specialists are scarce—provided deployment includes local validation and oversight [42].

### 6.5. Privacy, Security, and Medico-Legal Accountability

Imaging AI depends on data flows that create privacy and security obligations: patient images and report text may be transmitted to cloud services, logged by vendors, or reused for model improvement. Proposed ethical frameworks for clinical imaging AI emphasize transparency about data use, respect for patient autonomy, fair benefit-sharing, and clear governance of secondary use [43]; WHO guidance assigns governments and providers responsibility for data protection and calls for transparency about where and how patient data are processed [7,8]. Practical duties at the departmental level include using only institutionally approved, contractually governed tools for any patient-identifiable data; never entering identifiable patient information into unapproved consumer chatbots; and understanding where AI-processed data are stored and for what purposes they may be reused [8,43].

Accountability, finally, cannot be delegated to software. Legal and professional analyses concur that responsibility for the clinical report rests with the humans who produce and sign it, but which human—interpreting physician, reporting sonographer, or both—and what must be documented are determined by the scope-of-practice, professional, and legal frameworks of each jurisdiction reviewed in Section 3 [9,10,13]. The universal principle is human accountability; its allocation is local. Documenting AI involvement in the examination—tool, version where known, and any deviation from or disagreement with AI output—both reflects emerging transparency expectations and protects patients and practitioners by making the human oversight chain auditable [6,17]. Where an AI suggestion is overridden, recording the reason contributes to local learning and to the post-market monitoring that regulators increasingly require [5,6,35].

## 7. Good-Practice Guidelines for Sonographers Using AI in Imaging Reporting

**Figure 1:**
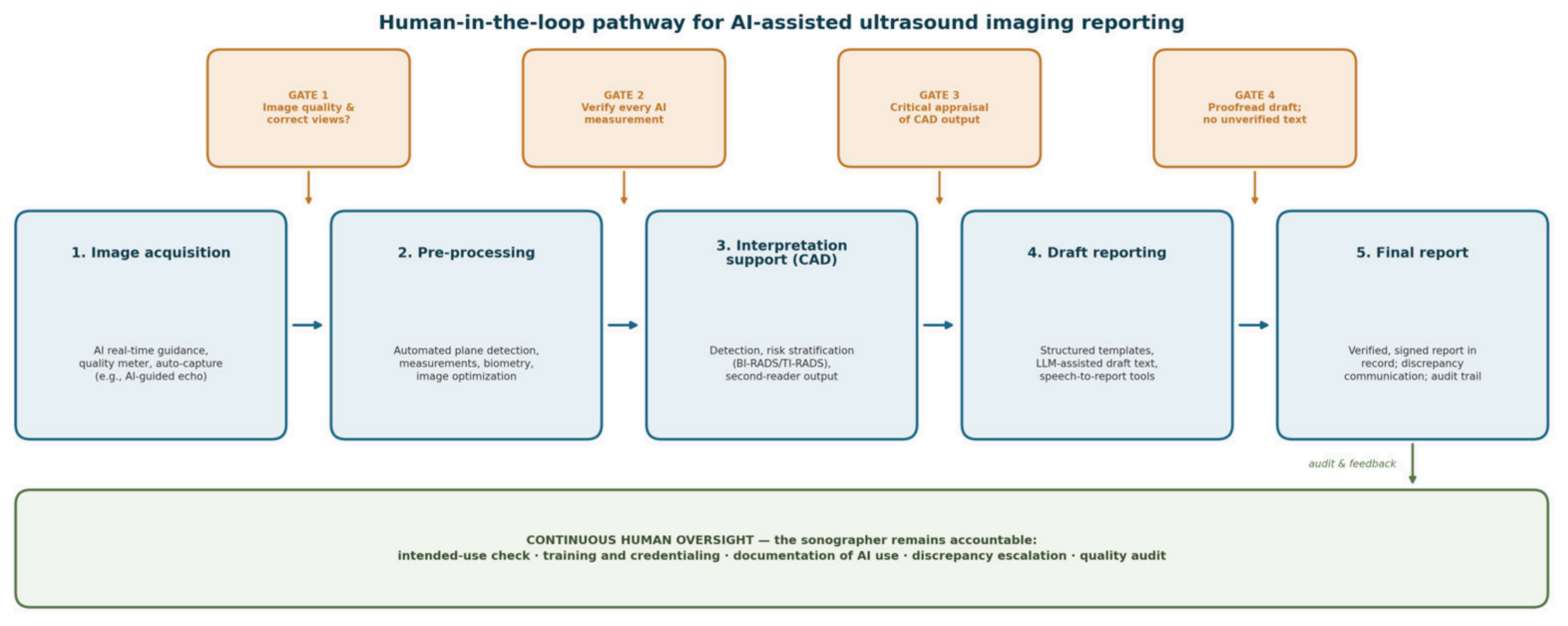
Human-in-the-loop pathway for AI-assisted ultrasound imaging reporting. AI tools support five stages from acquisition to the final signed report; at each stage a verification gate (Gates 1–4) keeps the sonographer in control, and a continuous oversight loop—spanning intended-use checks, training, documentation, escalation, and audit—closes the quality cycle. Blue boxes denote the five reporting-chain stages, orange boxes the sonographer verification gates, and the green box the continuous human-oversight loop. The pathway is a conceptual framework synthesized by the author from the evidence, guidelines, and regulations reviewed in this article; it has not been externally validated by stakeholder consultation, consensus methods, usability testing, or implementation studies, and should be regarded as a proposal for local adaptation and future evaluation.

The evidence and frameworks reviewed above converge on a coherent set of practical behaviors, organized here by phase of use and consolidated in Table 3 and Figure 1. Two qualifications apply to everything that follows. First, the recommendations and the human-in-the-loop pathway in Figure 1 constitute a framework synthesized by the author from the published evidence, consensus guidelines, and regulations reviewed in Section 4, Section 5 and Section 6; the framework has not undergone stakeholder review, Delphi consensus, usability testing, implementation testing, or validation by practicing sonographers, and it should be regarded as a proposed good-practice framework rather than an externally validated clinical guideline (see Section 9). Second, the recommendations do not share the same evidentiary basis: some restate regulatory or professional requirements, some translate consensus-framework recommendations, a few rest on direct clinical evidence—only some of it obtained in ultrasound—and others are author-derived good-practice recommendations. Table 3 therefore labels each recommendation by evidence category and by the level at which responsibility lies (individual sonographer, department/institution, manufacturer, regulator, or professional society), because items such as software-version control, cybersecurity, procurement validation, algorithm-drift monitoring, data-governance agreements, and post-market surveillance cannot be the responsibility of an individual sonographer. These recommendations are intended for adaptation into departmental protocols and are consistent with existing documentation standards, accreditation requirements, trustworthiness frameworks, and regulatory oversight obligations [6,9,10,17]; where they concern who may sign, who is accountable, or what must be recorded, they must be read in the light of jurisdiction-specific professional and legal requirements.

### 7.1. Before Use: Appraisal, Training, and Governance

Safe AI-assisted reporting starts before the first patient. Departments should confirm that each tool has appropriate regulatory clearance or marking for the specific intended use, clinical area, and user category, and should record what evidence supports it—using CLAIM/TRIPOD + AI-style questions about data provenance, external validation, and subgroup performance rather than relying on marketing summaries [5,6,29,30]. Sonographers should complete documented, tool-specific training and credentialing before independent use, mirroring established accreditation expectations for personnel competence [11], and should know the tool’s labeled limitations (patient populations, view types, contraindications) as well as its headline performance [15]. Governance arrangements are a departmental and institutional responsibility and should be in place before deployment: a named clinical lead, a local validation or pilot plan consistent with DECIDE-AI principles, procurement validation and data-governance agreements with the manufacturer, policies for software-version control and cybersecurity, a plan for monitoring algorithm drift, and a channel for reporting problems that feeds the manufacturer’s and regulator’s post-market surveillance [5,17,35]. The individual sonographer’s duty in this phase is to use only approved tools, to know the tool’s intended use and limitations, to complete the required training, and to use the reporting channel.

Equally important is clarity about roles. Protocols should state explicitly which outputs sonographers may act on, which require physician review, and what must be escalated; they should define who verifies automated measurements and AI-drafted text; and they should establish that AI use never extends a sonographer’s scope of practice beyond the professional standards and legal requirements applicable in that jurisdiction—whether physician-signed reporting, credentialed sonographer reporting, or another local model [10,13]. Transparency toward patients—informing them, where local policy requires, that AI assists parts of the examination—supports the autonomy and trust principles of WHO guidance and emerging regulation [6,7].

### 7.2. During the Examination and AI Use

During scanning, the governing principle is human-in-the-loop operation with verification at defined checkpoints (Figure 1). Input quality comes first: AI outputs are only as reliable as the views provided, so acquisition must follow protocol with correctly labeled, diagnostically adequate images, and AI quality indicators should prompt—not replace—sonographer judgment about clinical adequacy [10,15]. Every automated measurement should be verified on the image before acceptance: correct caliper placement, correct structure and phase, plausible value, with manual re-measurement whenever placement is doubtful [17]. CAD suggestions should be appraised critically as one item of evidence among the real-time findings, clinical context, and prior imaging; neither acceptance nor rejection should be automatic, and particular caution is warranted when the suggestion is conveniently reassuring, given documented discriminative automation bias [18,38]. When sonographers disagree with an AI output, they should trust their trained judgment, acquire additional views or repeat measurements, and escalate according to protocol rather than deferring to the algorithm by default [6,38].

Contemporaneous documentation protects quality and traceability. The technical record should note AI tool use as locally required, capture any AI-related limitation of the examination (for example, views the guidance software could not optimize), and flag discrepancies between AI output and sonographer assessment for the interpreting physician [10,17]. For generative drafting, the hard rule applies throughout: nothing enters the report that has not been verified against the images and measurements—numbers, laterality, findings, comparisons, and recommendations alike [8,28].

### 7.3. Reporting, Documentation, and Post-Examination Responsibilities

The final report remains a human product. Whether authored by a reporting sonographer within an approved scope or assembled for physician sign-off, it must meet documentation standards for content and terminology, accurately reflect the examination performed, and use standardized lexicons (for example, BI-RADS or TI-RADS) where applicable [10]. Any preliminary report issued should follow departmental policy, and discrepancies between preliminary and final interpretations must be documented and communicated as standards require [10]. Where AI contributed materially to measurements, categorization, or drafting, recording that contribution—consistent with transparency and traceability principles—supports audit, medico-legal clarity, and post-market learning [6,17,43].

After the examination, sonographers contribute to the monitoring loop that trustworthy-AI frameworks require: reporting implausible or erroneous AI outputs through incident and governance channels, participating in periodic audit of AI performance and override patterns, and engaging in continuing education that maintains both unaided scanning competence and up-to-date AI literacy [9,17]. Post-deployment monitoring needs defined triggers as well as routine audit. Departments should specify in advance the events that require a formal reassessment—and, if necessary, suspension—of an AI tool: (i) a major software or model update, including updates released under a predetermined change control plan; (ii) a change of ultrasound equipment, transducer, or acquisition presets on which the tool operates; (iii) a substantial change in the patient population served (for example, extension to pediatric, obstetric, or high-body-mass-index patients, or a new referral pathway); (iv) an unexpected cluster of erroneous or implausible outputs, incident reports, or discrepancies with final interpretation; (v) deterioration in locally monitored performance indicators, such as the proportion of measurements re-measured manually, override rates, agreement with final reports, or turnaround time; and (vi) a manufacturer safety notice, regulatory action, or new published evidence of harm or bias. Reassessment should follow the same appraisal steps as the initial adoption (Section 7.1), and sonographers are usually the first to observe triggers (ii), (iv), and (v), which is why the reporting channel must be easy to use and closed-loop [5,6,17,35]. Feedback from frontline users is also the most credible input for procurement renewal, workflow refinement, and professional-society advocacy; the WFUMB call for implementation guidance will be answered effectively only if practicing sonographers document and communicate their experience [9].

### 7.4. Operational Rules: Graded Oversight, Verification, and Discrepancy Handling

The four classes of AI application reviewed in Section 4 carry different levels of risk and therefore warrant different degrees of human oversight; the following operational rules are author-derived good-practice recommendations (evidence category G in Table 3) intended to be adapted into local protocols. (1) AI-assisted acquisition guidance (lowest risk): the AI quality indicator informs but never certifies adequacy. Verification means that the sonographer confirms, view by view, that the required anatomy is displayed in the correct plane and that the image answers the clinical question; an auto-captured clip that does not meet protocol is discarded and re-acquired, and any view the guidance could not optimize is documented as a limitation. (2) Automated measurements (moderate risk): adequate verification means that, for every automated value entering the report, the sonographer has visually confirmed caliper or contour placement on the correct landmarks, the correct cardiac phase or fetal plane, and physiological plausibility against reference ranges and against prior studies where available. A measurement is rejected and repeated manually when placement is doubtful, when the value falls outside the plausible range, or when the automated and manual values differ by more than a locally defined tolerance (for example, a percentage of the measurement or the known inter-observer variability for that parameter); where an automated value is retained after manual confirmation, the confirmed value is what enters the report. (3) Computer-aided diagnosis (higher risk): the CAD category is one input to be weighed against the real-time findings, clinical context, and prior imaging. It should be rejected—that is, not transcribed and not used to alter the recommended management—when it conflicts with a clear sonographic finding, when the lesion or patient falls outside the tool’s validated population or intended use, or when image quality is inadequate. A CAD output that is more reassuring than the sonographer’s assessment should never lower the level of concern recorded without physician review. (4) Generative report drafting (highest risk): AI-drafted text is treated as an unverified draft in its entirety. Verification means a line-by-line comparison of every number, laterality, finding, comparison, and recommendation against the images, the measurement record, and the prior report; any sentence that cannot be traced to a verified source is deleted, and the draft as a whole is rejected if it contains fabricated measurements or findings, because such errors indicate that the remaining text cannot be trusted.

Discrepancies between an AI output and professional judgment should be handled by a fixed sequence rather than by default deference: first, re-check the input (view quality, labeling, calipers); second, repeat the acquisition or measurement manually; third, if the disagreement persists, record the sonographer’s finding as the primary result and the AI output as a flagged discrepancy; fourth, escalate to the interpreting physician or reporting lead according to protocol before the report is finalized. Each discrepancy should be documented contemporaneously in the technical record or worksheet with, at minimum, the tool and version where known, the AI output, the sonographer’s finding, the action taken (accepted, corrected, rejected, escalated), and the reason; where local policy permits, a brief statement of AI involvement in the signed report supports transparency. Aggregated discrepancy records are the raw material for the audits and reassessment triggers described in Section 7.3.

## 8. Education, Institutional Implementation, and the Role of Professional Societies

Individual behavior change will not be sufficient without educational and institutional scaffolding. Sonography curricula—undergraduate, postgraduate, and continuing professional development—should incorporate structured AI literacy: how machine-learning models are trained and validated; what sensitivity, specificity, calibration, and AUC mean in practice; how to interrogate an IFU and a regulatory summary; how automation bias arises and how to counteract it; and how to document AI involvement in reports [9,17]. Simulation-based training offers a particularly promising vehicle, allowing deliberate exposure to incorrect AI advice in a safe environment so that verification reflexes are trained rather than assumed. Assessment frameworks should certify not only AI-assisted performance but also unaided competence, reflecting the deskilling evidence reviewed in Section 6.2 and the stewardship recommendations of the WFUMB literature [9,38].

At the institutional level, implementation should follow the phased logic of DECIDE-AI: limited pilot deployment with prospective collection of human-factors data, structured user feedback, and explicit criteria for scale-up, followed by continuous post-deployment monitoring for drift and differential performance across patient subgroups [17,35]. Procurement processes should require evidence dossiers addressing CLAIM/TRIPOD + AI-style transparency questions, contractual clarity on data use and privacy, and vendor commitments on update notification and performance monitoring [29,43]. Multidisciplinary AI governance committees—ideally including a sonographer representative—should own policies on approved tools, intended uses, training requirements, documentation expectations, and incident pathways.

Professional societies have distinctive leverage. The 2026 WFUMB editorial calls on bodies including WFUMB, AIUM, EFSUMB, and the Society of Radiologists in Ultrasound to develop implementation, validation, and scope-of-practice guidance; to press journals for prospective, multi-site validation and subgroup reporting; to integrate AI literacy into education while preserving unaided competence; and to engage regulators on monitoring and transparency requirements [9]. Existing ultrasound standards provide ready anchors for such guidance: documentation parameters can be extended to specify AI-use notation in reports, accreditation standards can incorporate AI competency and governance criteria, and position statements can clarify medico-legal expectations for human oversight [10,11,12]. International coordination matters for equity as well: validated AI tools deployed with local oversight could partially mitigate the global shortage of sonography expertise, but only if professional guidance addresses low-resource deployment explicitly rather than leaving it to improvisation [7,42].

## 9. Limitations of This Review and Future Directions

This review has limitations that follow from its narrative design and that readers should weigh before adopting its recommendations. First, although the search was structured (Section 2, Supplementary Table S1), it was not exhaustive, was restricted to English-language sources, and was screened and selected by a single author; selection bias—the preferential inclusion of sources that supported the emerging framework—cannot be excluded, and no formal inter-rater check was possible. Second, the peer-reviewed evidence base is itself susceptible to publication bias toward positive AI-performance results, and no funnel-plot or similar assessment was feasible for a body of evidence that is not quantitatively poolable. Third, no formal risk-of-bias assessment was performed, and the sources range from prospective validation studies through retrospective reader studies, regulatory summaries, professional standards, consensus guidelines, reviews, editorials, and expert commentary; the recommendations therefore rest on unequal evidentiary strength, which Table 3 makes explicit but cannot eliminate. Fourth, direct ultrasound evidence is scarce for several of the risks discussed—automation bias, deskilling, and generative-report hallucination in particular—so parts of Section 6 are extrapolated from mammography, radiography, pathology, and general radiology reporting, and their applicability to real-time sonographer workflows remains to be demonstrated. Fifth, the proposed phase-based framework and human-in-the-loop pathway (Table 3, Figure 1) are the author’s synthesis; they have not undergone stakeholder review, Delphi consensus, usability testing, implementation testing, or validation by practicing sonographers, and their feasibility, acceptability, and effect on error rates are unknown. Sixth, scope-of-practice and medico-legal statements are drawn mainly from United States, United Kingdom, European, and international documents and may not represent other health systems. Finally, regulatory status and generative-AI capabilities are evolving rapidly; regulatory and professional documents are cited with access dates for this reason, and readers should verify current status before relying on any specific statement. Empirical validation of the framework—through consensus methods with sonographer, radiologist, educator, patient, and regulator stakeholders, followed by usability and implementation studies measuring verification behavior and error rates—is the most important priority for future research arising from this review.

Several developments will shape the next iteration of guidance for sonographers. Technically, the field is moving from single-task tools toward foundation models and large multi-modal models capable of handling image, text, and clinical context jointly; such systems may eventually draft preliminary structured reports directly from cine data, making verification workflows and hallucination safeguards even more central to reporting practice [8,28]. Regulatory science is adapting through predetermined change control plans, real-world performance monitoring, and—in the European context—harmonized standards under the AI Act that will operationalize human-oversight requirements; how these instruments perform in practice will determine how much verification burden falls on individual users versus institutional systems [5,6]. Research priorities identified across the literature include prospective multi-site trials with subgroup performance reporting, human-factors studies of sonographer–AI interaction in real workflows (including experiments on automation bias during real-time scanning), quantification of deskilling trajectories and effective countermeasures, ultrasound-specific evaluation of generative report drafting, and patient-facing research on transparency preferences [9,17,35].

Standardization of practice is the most urgent need. Candidate elements include a uniform notation for documenting AI involvement in imaging reports; agreed minimum verification steps for automated measurements and CAD outputs; competency frameworks for AI-assisted sonography; and shared registries of real-world AI performance contributed by ultrasound departments. Each is achievable within existing professional structures if societies act on the stewardship agenda already articulated in the literature [9,10,11]. The goal is not to slow adoption but to make adoption durable: tools that are verified, monitored, and embedded in trained human workflows are far more likely to deliver their promised efficiency and consistency gains than tools adopted uncritically [17,38].

## 10. Conclusions

Artificial intelligence is restructuring the ultrasound imaging reporting pathway from the transducer to the signed report. For sonographers, this transformation expands rather than diminishes professional responsibility: the sonographer becomes the primary verifier of AI outputs, the guarantor of input quality, and the first line of defense against automation bias, hallucinated content, and out-of-scope use. The current guideline landscape—research-reporting checklists, trustworthiness frameworks, WHO ethics guidance, and emerging regulation—provides the principles, but sonographer-specific operational guidance remains scarce. This review has distilled the available evidence—of unequal strength, and in places extrapolated from other imaging domains—into a proposed, not yet externally validated, set of practical, phase-based recommendations whose evidentiary basis and responsible level are made explicit in Table 3: before use, confirm clearance, appraise evidence, train, and govern; during use, secure input quality and verify every AI-derived value and suggestion; in reporting, verify all AI-drafted content, meet documentation standards, and record AI involvement; after use, report problems, participate in audit, and maintain both core skills and AI literacy. Professional societies, educators, and institutions must now convert these principles into enforceable standards, curricula, and governance structures adapted to each jurisdiction’s scope-of-practice and legal framework, and researchers should test the proposed framework with practicing sonographers before it is adopted as a standard. Used with disciplined human oversight, AI can make ultrasound reporting more consistent, efficient, and accessible; used uncritically, it can propagate error at scale. The difference lies in the hands—and the training—of the sonographer.

## Figures and Tables

**Table 1:** Representative artificial intelligence (AI) applications across the ultrasound imaging–reporting chain, supporting evidence, and practical cautions for sonographers.

Reporting-Chain Stage	Example Tools/Functions	Key Evidence	Practical Cautions
Image acquisition	Real-time transducer guidance, quality meter, auto-capture (e.g., AI-guided echocardiography)	Novice-obtained scans diagnostic for primary cardiac assessments in 98.8% of cases (RV size 92.5%) [14,15]	Cleared for specific views/users; view-specific weaknesses (e.g., IVC 57.5%); operator must confirm clinical adequacy [15]
Automated measurement	Fetal biometry, ejection fraction, nodule sizing, standard-plane detection	Cleared tools listed on FDA AI/ML device list; most evidence retrospective [4,5]	Verify caliper placement and plausibility; re-measure manually when doubtful; re-check after software updates [10,17]
Computer-aided diagnosis (CAD)	Breast (Koios DS, S-Detect) and thyroid risk stratification	Reader AUC 0.83→0.87 with decision support [18]; S-Detect AUC 0.83 vs. 0.74 for radiologists [19]; pooled sensitivity 0.82/specificity 0.86 [20]	Probabilistic second reader, not a diagnosis; benefit varies with reader experience; generalizability limits [18,21,23]
Generative report drafting	LLM-based structuring of findings, error checking, impression drafting	Technical feasibility only: free-text→structured conversion on curated radiology reports [25]; retrospective error detection [26]; board-style exam benchmark [27]; no ultrasound-specific or prospective clinical-utility evidence	Hallucination risk; line-by-line verification mandatory; no unverified content in signed reports [8,28]

AI: artificial intelligence; AUC: area under the curve; CAD: computer-aided detection and diagnosis; FDA: United States Food and Drug Administration; IVC: inferior vena cava; LLM: large language model; ML: machine learning; RV: right ventricular.

**Table 2:** Major guidelines, frameworks, and regulations relevant to AI use in imaging reporting, and their practical relevance to sonographers.

Guideline/Framework (Year)	Type and Scope	Core Content	Relevance to Sonographers
CLAIM (2020) [29]	Reporting checklist for AI in medical imaging	Minimum study information: data, ground truth, model, evaluation	Appraising vendor evidence and study quality before adoption
TRIPOD + AI (2024) [30]	Reporting of prediction models (regression/ML)	Transparent description of data, participants, model, performance	Judging whether validation matches local population
STARD-AI (protocol 2021) [31]; QUADAS-AI (2021) [32]	Diagnostic accuracy reporting and bias assessment	AI-specific accuracy-study items; risk-of-bias domains	Critical reading of accuracy claims for CAD tools
SPIRIT-AI/CONSORT-AI (2020) [33,34]	Trial protocol and trial reporting extensions	Algorithm version, input/output handling, human–AI interaction, error analysis	Understanding intended human role and evidence limits
DECIDE-AI (2022) [35]	Early live clinical evaluation of decision support	Human factors, workflow integration, user variability, deviation reasons	Structuring local pilot deployments and user feedback
FUTURE-AI (2025) [17]	International consensus on trustworthy, deployable health AI	Six principles, 30 practices across the AI lifecycle	Departmental policies: quality control, auditing, logging, training
WHO guidance (2021; 2024) [7,8]	Ethics and governance of health AI; large multi-modal models	Six ethical principles; 40+ recommendations; warns of automation bias	Ethical duties: autonomy, transparency, accountability, equity
FDA AI/ML device list; device pathways (ongoing) [5,14]	Device authorization (510(k), De Novo)	Cleared intended use, special controls, change-control plans	Verify clearance status and labeled intended users/uses
EU AI Act—Regulation (EU) 2024/1689 [6]	Horizontal AI regulation	High-risk obligations incl. Article 14 human oversight	Legal basis for the right and duty to oversee and override AI
AIUM documentation parameter and accreditation standards [10,11]	Professional practice standards	Required report elements; personnel, QA, timeliness	Report content, discrepancy documentation, credentialing
WFUMB stewardship editorial (2026) [9]	Professional position	Calls for society guidance, validation standards, AI literacy	Agenda for professional development and society engagement

AI: artificial intelligence; AIUM: American Institute of Ultrasound in Medicine; CAD: computer-aided detection and diagnosis; EU: European Union; FDA: United States Food and Drug Administration; ML: machine learning; QA: quality assurance; WFUMB: World Federation for Ultrasound in Medicine and Biology; WHO: World Health Organization. Full names of the guidelines and frameworks listed in the first column are given in Section 5 and in the Abbreviations list.

**Table 3:** Good-practice recommendations for sonographers using AI tools in imaging reporting, by phase of use.

Phase	Recommendation	Supporting Basis [Evidence Category; Responsible Level] a
Before use	Confirm regulatory clearance/marking and labeled intended use, user category, and limitations of each AI tool	[5,6,15]; R; D, I
Before use	Appraise supporting evidence with structured questions (data provenance, external/multi-site validation, subgroup performance)	[29,30,32]; C; D, I
Before use	Complete documented tool-specific training and credentialing; maintain unaided scanning competence	[9,11]; R, C; I, D, P
Before use	Ensure local governance: clinical lead, pilot/validation plan, update and version policy, problem-reporting channel	[5,17,35]; C; D
During use	Guarantee input quality: protocol-based acquisition, correct labeling, adequate views before invoking AI	[10,15]; R, E-US; I
During use	Verify every automated measurement on the image (calipers, structure, phase, plausibility); re-measure when doubtful	[17]; C, G; I
During use	Appraise CAD output critically as one item of evidence; beware automation bias, especially reassuring suggestions	[18,38,39]; E-US [18], E-X [38,39]; I
During use	Escalate disagreement with AI per protocol; document AI-related examination limitations	[6,10]; R, G; I, D
During use	Treat LLM-drafted text as unverified draft; check all values, laterality, findings, and comparisons	[8,28]; C, G; I
Reporting	Ensure the signed report meets documentation standards and reflects the actual examination; use standardized lexicons	[10]; R; I
Reporting	Document material AI contributions and any overrides, with reasons, per local policy and jurisdictional documentation requirements	[6,17,43]; C, G; I, D
After use	Report implausible/erroneous AI outputs; participate in audit of AI performance and override patterns; support reassessment of the tool when a defined trigger occurs (see Section 7.3)	[17,35]; C, G; I, D, M, Rg
After use	Maintain continuing education in both core sonography skills and AI literacy	[9,11]; R, C; I, D, P

AI: artificial intelligence; CAD: computer-aided detection and diagnosis; LLM: large language model. a, Evidence category: R = regulatory or professional requirement; C = consensus-framework recommendation (e.g., FUTURE-AI, DECIDE-AI, WHO); E-US = direct clinical evidence obtained in ultrasound; E-X = clinical evidence extrapolated from other imaging or clinical domains; G = author-derived good-practice recommendation without direct empirical evidence in sonographers. Responsible level: I = individual sonographer; D = department/institution; M = manufacturer; Rg = regulator; P = professional society. Recommendations coded R or G are subject to jurisdiction-specific scope-of-practice and documentation rules; only the safety principles (verification, human oversight, escalation) are intended as universal.

## Data Availability

No new data were created or analyzed in this study. Data sharing is not applicable to this article.

## Supplementary Materials

The following supporting information can be downloaded at: https://www.mdpi.com/article/10.3390/healthcare14203548/s1, Table S1: Database-specific search strategies used for the narrative review.

## Abbreviations

The following abbreviations are used in this manuscript:

AI	Artificial intelligence
AIUM	American Institute of Ultrasound in Medicine
AUC	Area under the receiver operating characteristic curve
BI-RADS	Breast Imaging Reporting and Data System
CAD	Computer-aided detection and diagnosis
CLAIM	Checklist for Artificial Intelligence in Medical Imaging
CONSORT-AI	Consolidated Standards of Reporting Trials–Artificial Intelligence extension
DECIDE-AI	Developmental and Exploratory Clinical Investigations of Decision support systems driven by Artificial Intelligence
EFSUMB	European Federation of Societies for Ultrasound in Medicine and Biology
EU	European Union
FDA	United States Food and Drug Administration
FUTURE-AI	Fairness, Universality, Traceability, Usability, Robustness, and Explainability in Artificial Intelligence
IFU	Instructions for use
ISUOG	International Society of Ultrasound in Obstetrics and Gynecology
IVC	Inferior vena cava
LLM	Large language model
ML	Machine learning
QUADAS-AI	Quality Assessment of Diagnostic Accuracy Studies–Artificial Intelligence
RV	Right ventricle
SPIRIT-AI	Standard Protocol Items: Recommendations for Interventional Trials–Artificial Intelligence extension
STARD-AI	Standards for Reporting of Diagnostic Accuracy Studies–Artificial Intelligence
TI-RADS	Thyroid Imaging Reporting and Data System
TRIPOD + AI	Transparent Reporting of a multivariable prediction model for Individual Prognosis Or Diagnosis–Artificial Intelligence
WFUMB	World Federation for Ultrasound in Medicine and Biology
WHO	World Health Organization

## References

[1] Topol E.J. (2019). High-performance medicine: The convergence of human and artificial intelligence. Nat. Med..

[2] Akkus Z., Cai J., Boonrod A., Zeinoddini A., Weston A.D., Philbrick K.A., Erickson B.J. (2019). A survey of deep-learning applications in ultrasound: Artificial intelligence–powered ultrasound for improving clinical workflow. J. Am. Coll. Radiol..

[3] Wang Y., Ge X., Ma H., Qi S., Zhang G., Yao Y. (2021). Deep learning in medical ultrasound image analysis: A review. IEEE Access.

[4] Fiorentino M.C., Villani F.P., Di Cosmo M., Frontoni E., Moccia S. (2023). A review on deep-learning algorithms for fetal ultrasound-image analysis. Med. Image Anal..

[5] U.S. Food and Drug Administration Artificial Intelligence and Machine Learning (AI/ML)-Enabled Medical Devices. https://www.fda.gov/medical-devices/software-medical-device-samd/artificial-intelligence-and-machine-learning-aiml-enabled-medical-devices.

[6] European Parliament and Council of the European Union (2024). Regulation (EU) 2024/1689 of the European Parliament and of the Council of 13 June 2024 Laying Down Harmonised Rules on Artificial Intelligence (Artificial Intelligence Act). Off. J. Eur. Union.

[7] World Health Organization (2021). Ethics and Governance of Artificial Intelligence for Health: WHO Guidance.

[8] World Health Organization (2024). Ethics and Governance of Artificial Intelligence for Health: Guidance on Large Multi-Modal Models (LMMs).

[9] Dighe M. (2026). Artificial intelligence in ultrasound imaging: Inevitable, promising, and in need of responsible stewardship. WFUMB Ultrasound Open.

[10] American Institute of Ultrasound in Medicine (2020). AIUM practice parameter for documentation of an ultrasound examination. J. Ultrasound Med..

[11] American Institute of Ultrasound in Medicine Standards and Guidelines for the Accreditation of Ultrasound Practices. https://www.aium.org/resources/official-statements/view/standards-and-guidelines-for-the-accreditation-of-ultrasound-practices.

[12] Jenssen C., Gilja O.H., Serra A.L., Piscaglia F., Dietrich C.F., Rudd L., Sidhu P.S. (2019). European Federation of Societies for Ultrasound in Medicine and Biology (EFSUMB) Policy Document Development Strategy—Clinical practice guidelines, position statements and technological reviews. Ultrasound Int. Open.

[13] Society and College of Radiographers, British Medical Ultrasound Society (2008). Guidelines for Professional Working Standards: Ultrasound Practice.

[14] U.S. Food and Drug Administration (2020). De Novo Classification Request for Caption Guidance: Decision Summary (DEN190040).

[15] Narang A., Bae R., Hong H., Thomas Y., Surette S., Cadieu C., Chaudhry A., Martin R.P., McCarthy P.M., Rubenson D.S. (2021). Utility of a deep-learning algorithm to guide novices to acquire echocardiograms for limited diagnostic use. JAMA Cardiol..

[16] Ossaba S., Diez Á., Marti M., Parra-Gordo M.L., Alonso-Gonzalez R., Tenajas R., Garzón G. (2024). Validation of a deep-learning modular prototype to guide novices to acquire diagnostic ultrasound images from urinary system. WFUMB Ultrasound Open.

[17] Lekadir K., Frangi A.F., Porras A.R., Glocker B., Cintas C., Langlotz C.P., Weicken E., Asselbergs F.W., Prior F., Collins G.S. (2025). FUTURE-AI: International consensus guideline for trustworthy and deployable artificial intelligence in healthcare. BMJ.

[18] Mango V.L., Sun M., Wynn R.T., Ha R. (2020). Should we ignore, follow, or biopsy? Impact of artificial intelligence decision support on breast ultrasound lesion assessment. Am. J. Roentgenol..

[19] Zhao C., Xiao M., Ma L., Ye X., Deng J., Cui L., Guo F., Wu M., Luo B., Chen Q. (2022). Enhancing performance of breast ultrasound in opportunistic screening women by a deep learning-based system: A multicenter prospective study. Front. Oncol..

[20] Li J., Sang T., Yu W.H., Jiang M., Hunag S.Y., Cao C.L., Chen M., Cao Y.W., Cui X.W., Dietrich C.F. (2020). The value of S-Detect for the differential diagnosis of breast masses on ultrasound: A systematic review and pooled meta-analysis. Med. Ultrason..

[21] Wang X.Y., Cui L.G., Feng J., Chen W. (2021). Artificial intelligence for breast ultrasound: An adjunct tool to reduce excessive lesion biopsy. Eur. J. Radiol..

[22] Wildman-Tobriner B., Taghi-Zadeh E., Mazurowski M.A. (2022). Artificial intelligence (AI) tools for thyroid nodules on ultrasound, from the *AJR* special series on AI applications. Am. J. Roentgenol..

[23] Fruchtman Brot H., Mango V.L. (2024). Artificial intelligence in breast ultrasound: Application in clinical practice. Ultrasonography.

[24] Chen P., Tong J., Lin T., Wang Y., Yu Y., Chen M., Yang G. (2022). The added value of S-Detect in the diagnostic accuracy of breast masses by senior and junior radiologist groups: A systematic review and meta-analysis. Gland Surg..

[25] Adams L.C., Truhn D., Busch F., Kader A., Niehues S.M., Makowski M.R., Bressem K.K. (2023). Leveraging GPT-4 for post hoc transformation of free-text radiology reports into structured reporting: A multilingual feasibility study. Radiology.

[26] Gertz R.J., Dratsch T., Bunck A.C., Lennartz S., Iuga A.I., Hellmich M.G., Persigehl T., Pennig L., Gietzen C.H., Fervers P. (2024). Potential of GPT-4 for detecting errors in radiology reports: Implications for reporting accuracy. Radiology.

[27] Bhayana R., Krishna S., Bleakney R.R. (2023). Performance of ChatGPT on a radiology board-style examination: Insights into current strengths and limitations. Radiology.

[28] Bhayana R. (2024). Chatbots and large language models in radiology: A practical primer for clinical and research applications. Radiology.

[29] Mongan J., Moy L., Kahn C.E. (2020). Checklist for Artificial Intelligence in Medical Imaging (CLAIM): A guide for authors and reviewers. Radiol. Artif. Intell..

[30] Collins G.S., Moons K.G.M., Dhiman P., Riley R.D., Beam A.L., Van Calster B., Ghassemi M., Liu X., Reitsma J.B., van Smeden M. (2024). TRIPOD+AI statement: Updated guidance for reporting clinical prediction models that use regression or machine learning methods. BMJ.

[31] Sounderajah V., Ashrafian H., Golub R.M., Shetty S., De Fauw J., Hooft L., Moons K., Collins G., Moher D., Bossuyt P.M. (2021). Developing a reporting guideline for artificial intelligence-centred diagnostic test accuracy studies: The STARD-AI protocol. BMJ Open.

[32] Sounderajah V., Ashrafian H., Rose S., Shah N.H., Ghassemi M., Golub R., Kahn C.E., Esteva A., Karthikesalingam A., Mateen B. (2021). A quality assessment tool for artificial intelligence-centered diagnostic test accuracy studies: QUADAS-AI. Nat. Med..

[33] Cruz Rivera S., Liu X., Chan A.W., Denniston A.K., Calvert M.J., SPIRIT-AI and CONSORT-AI Working Group (2020). Guidelines for clinical trial protocols for interventions involving artificial intelligence: The SPIRIT-AI extension. Nat. Med..

[34] Liu X., Cruz Rivera S., Moher D., Calvert M.J., Denniston A.K., SPIRIT-AI and CONSORT-AI Working Group (2020). Reporting guidelines for clinical trial reports for interventions involving artificial intelligence: The CONSORT-AI extension. Nat. Med..

[35] Vasey B., Nagendran M., Campbell B., Clifton D.A., Collins G.S., Denaxas S., Denniston A.K., Faes L., Geerts B.F., Ibrahim M. (2022). Reporting guideline for the early-stage clinical evaluation of decision support systems driven by artificial intelligence: DECIDE-AI. Nat. Med..

[36] European Society of Radiology (ESR) (2019). What the radiologist should know about artificial intelligence—An ESR white paper. Insights Imaging.

[37] Vollmer S., Mateen B.A., Bohner G., Király F.J., Ghani R., Jonsson P., Cumbers S., Jonas A., McAllister K.S.L., Myles P. (2020). Machine learning and artificial intelligence research for patient benefit: 20 critical questions on transparency, replicability, ethics, and effectiveness. BMJ.

[38] Dratsch T., Chen X., Rezazade Mehrizi M., Kloeckner R., Mähringer-Kunz A., Püsken M., Baeßler B., Sauer S., Maintz D., Pinto dos Santos D. (2023). Automation bias in mammography: The impact of artificial intelligence BI-RADS suggestions on reader performance. Radiology.

[39] Gaube S., Suresh H., Raue M., Merritt A., Berkowitz S.J., Lermer E., Coughlin J.F., Guttag J.V., Colak E., Ghassemi M. (2021). Do as AI say: Susceptibility in deployment of clinical decision-aids. npj Digit. Med..

[40] Kiani A., Uyumazturk B., Rajpurkar P., Wang A., Gao R., Jones E., Yu Y., Langlotz C.P., Ball R.L., Montine T.J. (2020). Impact of a deep learning assistant on the histopathologic classification of liver cancer. npj Digit. Med..

[41] Challen R., Denny J., Pitt M., Gompels L., Edwards T., Tsaneva-Atanasova K. (2019). Artificial intelligence, bias and clinical safety. BMJ Qual. Saf..

[42] Berg W.A., López Aldrete A.L., Jairaj A., Ledesma Parea J.C., García C.Y., McClennan R.C., Cen S.Y., Larsen L.H., de Lara M.T.S., Love S. (2023). Toward AI-supported US triage of women with palpable breast lumps in a low-resource setting. Radiology.

[43] Larson D.B., Magnus D.C., Lungren M.P., Shah N.H., Langlotz C.P. (2020). Ethics of using and sharing clinical imaging data for artificial intelligence: A proposed framework. Radiology.

