# Supplementary material for: Responsible Use of Artificial Intelligence Tools in Imaging Reporting: Practical Guidelines for Sonographers—A Narrative Review
Source: Healthcare (Basel). 2026 Oct 21;14(20):3548. doi: 10.3390/healthcare14203548 (PMC13704229; doi:10.3390/healthcare14203548)
Supplement: Supplementary file 1 [file healthcare-14-03548-s001.zip › healthcare-4522321-supplementary.pdf]

Supplementary Table S1. Database-specific search strategies used for the narrative review.

Search period: 1 January 2015 to 31 July 2026. Language: English. All searches were performed by the sole author on 18 July 2026. Records were exported to EndNote, duplicates were removed automatically and confirmed manually, and titles/abstracts and then full texts were screened by the sole author against the eligibility criteria stated in Section 2 of the main text.

Database / source	Search string (as entered)	Filters / limits	Search date	Records retrieved
PubMed/MEDLINE	(("artificial intelligence"[Title/Abstract] OR "deep learning"[Title/Abstract] OR "machine learning"[Title/Abstract] OR "large language model*"[Title/Abstract] OR "Artificial Intelligence"[Mesh] OR "Deep Learning"[Mesh] OR "Machine Learning"[Mesh]) AND (ultrasound[Title/Abstract] OR ultrasonograph*[Title/Abstract] OR sonograph*[Title/Abstract] OR sonographer*[Title/Abstract] OR echocardiograph*[Title/Abstract] OR "Ultrasonography"[Mesh]) AND ("imaging report*"[Title/Abstract] OR "structured report*"[Title/Abstract] OR "radiology report*"[Title/Abstract] OR "automation bias"[Title/Abstract] OR deskilling[Title/Abstract] OR guideline*[Title/Abstract] OR "human oversight"[Title/Abstract] OR "computer-aided"[Title/Abstract] OR "decision support"[Title/Abstract]))	Publication date 2015/01/01–2026/07/31; English; humans	18 July 2026	612
Scopus	TITLE-ABS-KEY(("artificial intelligence" OR "deep learning" OR "machine learning" OR "large language model*") AND (ultrasound OR ultrasonograph* OR sonograph* OR sonographer* OR echocardiograph*) AND ("imaging report*" OR "structured report*" OR "radiology report*" OR "automation bias" OR deskilling OR guideline* OR "human oversight" OR "computer-aided" OR "decision support"))	PUBYEAR > 2014 AND PUBYEAR < 2027; LANGUAGE(English); DOCTYPE(ar OR re OR ed OR le)	18 July 2026	894
Google Scholar	Query 1: "artificial intelligence" sonographer "imaging reporting"<br>Query 2: "large language model" ultrasound report<br>Query 3: "automation bias" ultrasound OR sonography<br>Query 4: deskilling ultrasound "artificial intelligence"<br>Query 5: guideline "artificial intelligence" sonography reporting	Custom range 2015–2026; first 200 records per query, sorted by relevance; patents and citations excluded	18 July 2026	1,000 screened (5 × 200); 332 unique after removal of duplicates with PubMed/Scopus records
Professional-body and regulatory websites (AIUM, WFUMB, EFSUMB, ISUOG, SoR/BMUS, US FDA, EU/EUR-Lex, WHO)	Site search and manual browsing of official statements, practice parameters, accreditation standards, guidance, device lists, and regulations using the terms “artificial intelligence,” “AI,” “machine learning,” “documentation,” “reporting,” “sonographer”	Most recent version of each document; access dates recorded in the reference list (17 July 2026)	17 July 2026	7 documents retained (references 5, 6, 7, 8, 11, 13, 14)
Hand-searching of reference lists of key papers	Reference lists of included reviews, guidelines, and editorials (e.g., references 4, 9, 17, 23, 28) screened manually	—	18–20 July 2026	2 additional sources retained
Total				2,506 records retrieved; 1,838 unique after duplicate removal; 1,710 excluded at title/abstract; 128 full texts assessed; 85 excluded at full text; 43 sources retained and cited (34 from

				database searches, 9 from websites and hand-searching)

Reasons for exclusion at full text (n = 85): AI application outside medical imaging or outside ultrasound with no reporting relevance (n = 41); technical model-development papers without user, reporting, or governance content (n = 27); superseded versions of guidelines or regulatory documents (n = 9); not available in English (n = 5); duplicate publications of the same dataset (n = 3). AIUM: American Institute of Ultrasound in Medicine; BMUS: British Medical Ultrasound Society; EFSUMB: European Federation of Societies for Ultrasound in Medicine and Biology; EU: European Union; FDA: Food and Drug Administration; ISUOG: International Society of Ultrasound in Obstetrics and Gynecology; SoR: Society and College of Radiographers; WFUMB: World Federation for Ultrasound in Medicine and Biology; WHO: World Health Organization.
